# *Tp53* Mutation Inhibits Ubiquitination and Degradation of WISP1 via Down-Regulation of Siah1 in Pancreatic Carcinogenesis

**DOI:** 10.3389/fphar.2018.00857

**Published:** 2018-08-03

**Authors:** Wei Wu, Xu Liu, Lumin Wei, Tong Li, Yi Zang, Yuting Qian, Tingting Bai, Juanjuan Li, Mingping Xie, Ying Zhu, Qi Wang, Lifu Wang

**Affiliations:** Department of Gastroenterology, Ruijin Hospital Affiliated to The Shanghai Jiao Tong University School of Medicine, Shanghai, China

**Keywords:** WISP1, *Tp53* mutation, Siah1, pancreatic cancer, carcinogenesis

## Abstract

Wnt1 inducible signaling pathway protein-1 (WISP1) may play an important role in promoting carcinogenesis. However, the biological function and underlying mechanism of WISP1 in pancreatic carcinogenesis still remains enigmatic. In this study, immunochemistry staining showed that protein levels of WISP1 were more significantly upregulated in pancreatic ductal adenocarcinoma (PDAC) tissues with *Tp53* mutation than in PDAC tissues with *Tp53* wild-type. In addition, a significant correlation was observed between increased malignant phenotype of tumors from well-differentiated adenocarcinoma tissues to moderately- or poorly-differentiated adenocarcinoma tissues shifting from cytoplasmic expression to nuclear accumulation of WISP1. Interestingly, *WISP1* expression was correlated with the poor prognosis in PDAC patients with *Tp53* mutation. Also, the biological function analysis showed that *WISP1* may act as a potential oncogene in PDAC cells. In addition, immunofluorescence analysis showed that *Tp53* mutation promoted *WISP1* expression in PanIN and PDAC cells, while Siah E3 Ubiquitin Protein Ligase 1 (*Siah1*) inhibited *WISP1* expression in PDAC cells. Moreover, through immunoprecipitation, immunoblotting analysis, *in vitro* binding assay, and ubiquitination assay, we found that *Tp53* mutation inhibited ubiquitination and degradation of Siah1-dependent WISP1. Therefore, *Tp53* mutation-Siah1-WISP1 is a new signaling pathway, playing an important role in pancreatic carcinogenesis.

## Introduction

Pancreatic ductal adenocarcinoma is one of the most malignant tumors of the gastrointestinal tract and its incidence grows with the social and economic development levels. In spite of continuous efforts on its early diagnosis and treatment, in the recent 5 years, the survival rate of pancreatic cancer still remains as low as 9% (Siegel et al., 2018). The known suppressors *Tp53*, *Smad4*, and *p16* are frequently inactivated in PDAC. *Tp53* mutation is detected in 50–70% of PDAC patients (Rosenfeldt et al., 2013), disturbing normal cell functions. Wnt signaling pathway is highly conservative and its relevant mutations are universal among PDAC patients (Jones et al., 2008). Our previous study has also showed a correlation between *Tp53* mutation and WISP1 (Wang et al., 2015). WISP1 is a matricellular protein and plays a significant role in regulation of cellular signaling networks (Berschneider and Konigshoff, 2011). Recently, abnormal expression of WISP1 has been proven in various types of human malignancies (Gurbuz and Chiquet-Ehrismann, 2015; Chahal et al., 2016; Wu et al., 2016; Jing et al., 2017). A previous study demonstrated that WISP1 protects human lung and breast cancer cells from p53-dependent cell death, suggesting that there could be a crosstalk between Tp53 and WISP1 signaling pathways (Su et al., 2002). Nevertheless, the mechanism behind remains unknown.

Recently, several studies showed that Tp53 may promote Siah1 protein levels, which is an E3 ubiquitin-protein ligase that mediates ubiquitination and subsequent proteasomal degradation of target proteins (Fujita et al., 2010; Yuan et al., 2017). These findings motivated us to examine whether E3 is an ubiquitin ligase SIAH1 mediates ubiquitination and degradation of WISP1. In our study, WISP1 was probably an oncogene, and its protein level was observed more significant for upregulation in PDAC tissues and PDAC cells with *Tp53* mutation than in PDAC tissues and PDAC cells with *Tp53* wild-type. Moreover, we attempted to demonstrate that *Tp53* mutation may downregulate Siah1 protein levels, which may inhibit ubiquitination and degradation of Siah1-dependent WISP1 and induce WISP1 nuclear import.

## Materials and Methods

### Patients and Tissue Samples

In this study, 203 PDAC and paraneoplastic tissues post operation were retrospectively obtained from Ruijin Hospital (Shanghai, China) before 2017. The consent of participants was obtained for PDAC research. None of the patients had undergone radiotherapy or chemotherapy before surgery. The tissues were embedded in paraffin wax for analysis. Histological diagnoses were performed by two independent senior pathologists. This study was carried out in accordance with the recommendations of the Ethics Committee of Ruijin Hospital, affiliated with Shanghai Jiao Tong University, School of Medicine with written informed consent from all subjects. All subjects gave written informed consent in accordance with the Declaration of Helsinki. The protocol was approved by the Ethics Committee of Ruijin Hospital, affiliated with Shanghai Jiao Tong University, School of Medicine.

### Cell Lines

Low-passage-number cells (P8) of the preinvasive pancreatic ductal cell line SH-PAN isolated from *Pdx-1-Cre; LSL-Kras^*G12D*/+^* mutant mice was employed. The SH-PAN cell line has only *Kras* mutation (Hingorani et al., 2003, 2005). Human PDAC cell lines with wild-type *Tp53* (Capan-2, HPAC) and *Tp53* mutants (Panc-1, MIA PaCa-2, HPAF-II-1, BxPC-3, AsPC-1), were purchased from the American Type Culture Collection (Sipos et al., 2003; Deer et al., 2010). Pancreatic carcinoma cell lines were cultured in Dulbecco’s modified Eagle’s medium (DMEM) (Panc-1, HPAC, HPAF-II), RPMI-1640 medium (AsPC-1 and BxPC-3), McCoy’s 5a medium (MIA PaCa-2), and Iscove’s Modified Dulbecco’s medium (Capan-2). All cells cultured in the abovementioned media were supplemented with 10% heat-inactivated fetal bovine serum (FBS) at 37°C with 5% CO_2_.

### Reagents

MG132 (Proteasome inhibitor), Cycloheximide (inhibitor of protein synthesis in eukaryotes), and Nutlin-3a (inhibitor of the MDM2-p53 interaction) were purchased from Sigma-Aldrich (St. Louis, MO, United States).

### Plasmid Constructs and Lentivirus-Mediated shRNA or Gene Overexpression

The shRNA target sequences containing four different sequences of humanWISP1 (GenBank accession number: NM_003882.3) and human Siah1 (GenBank accession number: NM_003031.3) were selected for shRNA interference. The mouse Tp53 (GenBank accession number: NM_011640.3) cDNA fragment was polymerase chain reaction PCR-amplified and mutated in *Tp53^*R172H*^*. The human *Tp53* (GenBank accession number: NM_001276760.1) cDNA fragment was PCR-amplified and mutated in *Tp53^*R175H*^* or *Tp53^*R273H*^*. Lentivirus-mediated overexpression of the mouse *Tp53^*R172H*^*, the human *Tp53^*R175H*^*, the human *Tp53^*R273H*^*, the human wild-type *Tp53*, and the mouse WISP1 (GenBank accession number: NM_018865.2) was constructed using the pGLV5-EF1a-GFP vectors (GenePharma, Shanghai, China). The full-length human Siah-1 cDNA was cloned into pGEX-KG (American Type Culture Collection, Manassas, VA, United States) to generate *E. coli* expression constructs of GST-Siah-1 fusion proteins. For synthesis of *in vitro* protein, the full-length WISP1 cDNA was cloned into pEXP1-DEST vector (Invitrogen, Carlsbad, CA, United States) to produce a polyhistidine tagged WISP1 (His6-WISP1) and a polypeptide protein tagged WISP1(Myc-WISP1). The cDNA fragment for GST-Siah-1 fusion protein (full-length, wild-type Siah-1; as in pGEX-KG) was also cloned in pEXP1-DEST. pRK5-HA-Ub for overexpression of HA-tagged ubiquitin was previously described (Conze et al., 2008). All constructs were verified by DNA sequencing. The lentivirus vector transduction was performed as previously described (Wang et al., 2016). Transient transfection of plasmid DNA was performed using Lipofectamine 2000 transfection reagent (Invitrogen). Cells were grown in culture medium with 10 μg/ml puromycin for selection of stable cells (Wang et al., 2013, 2016). Primers used are shown in **Table 1**.

**Table 1:** Primers used in this study.

Name	
Siah1 shRNA (human)	
SR-1F	5′-GATCCGTCTTAGAGAAACAGGAAATTCAAGAGATTTCCTGTTTCTCTAAGACTTTTTTG-3′
SR-1R	5′-AATTCAAAAAAGTCTTAGAGAAACAGGAAATCTCTTGAATTTCCTGTTTCTCTAAGACG-3′
SR-2F	5′-GATCCAAGGAATTGCAACAGCCATTCAAGAGATGGCTGTTGCAATTCCTT TTTTTTG-3′
SR-2R	5′-AATTCAAAAAA AAGGAATTGCAACAGCCATCTCTTGAATGGCTGTTGCAATTCCTT G-3′
SR-3F	5′-GATCC AGTAAACCACTGAAAAAATTCAAGAGA TTTTTTCAGTGGTTTACT TTTTTTG-3′
SR-3R	5′-AATTCAAAAAA AGTAAACCACTGAAAAAATCTCTTGAA TTTTTTCAGTGGTTTACT G-3′
SR-4F	5′-GATCC AGTATCTTTTGATATGCTTCAAGAGA GCATATCAAAAGATACTTTTTTTG-3′
SR-4R	5′-AATTCAAAAAA AGTATCTTTTGATATGCTCTCTTGAA GCATATCAAAAGATACT G-3′
WISP1 shRNA (human)	
WR-1F	5′-GATCC TAGGAGTGTGTGCACAGGTTCAAGAGA CCTGTGCACACACTCCTA TTTTTTG-3′
WR-1R	5′-AATTCAAAAAA TAGGAGTGTGTGCACAGGTCTCTTGAA CCTGTGCACACACTCCTA G-3′
WR-2F	5′-GATCC GCACACGCTCCTATCAACTTCAAGAGA GTTGATAGGAGCGTGTGC TTTTTTG-3′
WR-2R	5′-AATTCAAAAAA GCACACGCTCCTATCAACTCTCTTGAA GTTGATAGGAGCGTGTGC G-3′
WR-3F	5′-GATCC GAAATGGAAT CAGGTAGATTCAAGAGA TCTACCTGATTCCATTTCTTTTTTG-3′
WR-3R	5′-AATTCAAAAAA GAAATGGAAT CAGGTAGATCTCTTGAA TCTACCTGATTCCATTTC G-3′
WR-4F	5′-GATCC CTCTTATAGT CTTTCTAGTTCAAGAGA CTAGAAAGACTATAAGAG TTTTTTG-3′
WR-4R	5′-AATTCAAAAAA CTCTTATAGT CTTTCTAGTCTCTTGAA CTAGAAAGACTATAAGAG G-3′
NC-F	5′-GATCCTTGCGCAACTGTGTCACGTTTCAAGAGAACGTGACACAGTTGCGCAATTTTTTG-3′
NC-R	5′-AATTCAAAAAATTGCGCAACTGTGTCACGTTCTCTTGAAACGTGACACAGTTGCGCAAG-3′

### Immunoprecipitation and Immunoblotting

Cells were lysed by sonicating for 5 s in 1 ml of detergent-free lysis buffer [phosphate buffered saline (PBS), 5 mM of Ethylenediaminetetraacetic acid (EDTA], 0.02% sodium azide, 10 mM of iodoacetamide, 1 mM of phenylmethane sulfonyl fluoride (PMSF), and 2 mg of leupeptin) at 4°C. Antibody-conjugated beads were prepared by combining 1 mg of antibodies with 30 ml of a 50% protein. A Sepharose bead slurry in 0.5 ml of ice-cold PBS was incubated for 1 h at 4°C in a tube rotator and were then washed three times with 1 ml of lysis buffer. The antibodies used for coimmunoprecipitation were Akt (ab6076, Abcam, Cambridge, United Kingdom). The beads were washed three times with washing buffer (50 mM of Tris-HCl, 300 mM of NaCl, 5 mM of EDTA, 0.02% sodium azide, and 0.1% Triton X-100) and once with ice-cold PBS. Immunodetection was carried out using the ECL Western Blotting Detection Kit (Amersham Corp., Burlington, MA, United States). Proteins were detected using antibodies against WISP1 (ab178547, Abcam, Cambridge, United Kingdom), wild-type *Tp53* (ab31333, Abcam, Cambridge, United Kingdom), Siah1 (ab2237, Abcam, Cambridge, United Kingdom), Myc tag (ab9106, Abcam, Cambridge, United Kingdom), HA tag (ab9110, Abcam, Cambridge, United Kingdom), and anti-GST (ab19256, Abcam, Cambridge, United Kingdom); and glyceraldehyde 3-phosphate dehydrogenase (GAPDH) levels were used as an internal reference control for the relative protein expression levels.

### *In Vitro* Binding Assay

*In vitro* protein synthesis of His6-tagged WISP1, GST and GST-Siah-1 fusion protein was performed using Expressway cell-free *E. coli* expression system (Invitrogen, Waltham, CA, United States), followed by purification of His6-tagged WISP1 using Ni-NTA magnetic agarose beads (Qiagen, München, Germany) and of GST and GST-Siah-1 using glutathione-Sepharose 4FF (GE Healthcare, Piscataway, NJ, United States). The eluted proteins were analyzed by immunoblot as previously described.

### Ubiquitination Assay

The cells were transfected with Siah-1 shRNA or control shRNA for 24 h, and then transfected with pEXP1-DEST-Myc-WISP1, pRK5-HA-Ub, and pGLV5-p53 plasmids. After 24 h, the cells were treated with 15 μM of MG132 for 8 h and lysed in the buffer containing 50 mM of 4-(2-hydroxyethyl)-1-piperazineethanesulfonic acid (HEPES, pH 7.5), 150 mM of NaCl, 1% Triton X-100, 1% glycerol, 10 μM of MG132, 100 mM of NaF, 1 mM of NaVO3, 25 mM of β-glycerophosphate, and complete protease inhibitors. Immunoprecipitation and immunoblot were performed as described above.

### Cell Proliferation, Soft Agar Assay, and Invasion Assay

For the cell proliferation assay, cells were seeded in a 96-well plate at a concentration of 5 × 103 cells/well 1 day before the experiment. 3-[4,5-Dimethylthiazol-2-yl]-2,5- diphenyltetrazolium bromide (MTT, 0.5 mg/ml, Sigma, St. Louis, MO, United States) was added to each well 1, 2, 3, 4, and 5 days after seeding. Cells were cultured at 37°C for 4 h, followed by addition of 150 μl of dimethylsulfoxide (DMSO). Absorption was measured at a wave length of 490 nm. For the soft agar assay, cells were seeded in six-well plates for the soft agar assay. Each well contained a bottom layer of 1% agarose, a middle layer of 0.5% agarose that included 1000 cells, and a top layer of medium, which was changed every 6 days. After 26 days, colonies were counted by Quantityone analysis software (BioRad Inc., Hercules, CA, United States). The transwell invasion assay was performed using a Millicell invasion chamber (Millipore, Burlington, MA, United States). The 8-μm pore inserts were coated with 15 μg of Matrigel (Becton Dickinson Labware, Bedford, MA, United States), and 5 × 10^4^ cells were seeded in the top chamber. The Matrigel invasion chamber was incubated for 20 h in a humidified tissue culture incubator. Non-invading cells were removed from the top of the Matrigel with a cotton-tipped swab. Invading cells on the bottom surface of the filter were fixed in methanol and stained with crystal violet. Invasion ability was determined by counting the stained cells (Wang et al., 2013, 2016).

### Nude Mouse Subcutaneous Xenograft and Metastasis Model

The BALB/c nude mice were divided into experimental group and control group. The cells (5 × 10^6^ per mouse) were injected into the subcutis of nude mice through five of experimental or control group, and 25 days later, tumor volume (V) was estimated using the formula V = LW^2^/6 (L: length of tumor; W: width of tumor). Also, the cells (1 × 10^6^ per mouse) were injected into the tail vein of nude mice in groups of five of experimental or control group, and 60 days later each mouse’s lung was removed and lung metastases were determined. This study was carried out in accordance with the recommendations of the Institutional Animal Care and Use Committee of the Shanghai Experimental Animals Centre of Chinese Academy of Sciences. The protocol was approved by the Institutional Animal Care and Use Committee of the Shanghai Experimental Animals Centre of Chinese Academy of Sciences.

### Histology, Immunohistochemistry, and Immunofluorescence

Four-micrometer-thick sections were stained with hematoxylin and eosin for histological verification. Primary antibodies against WISP1 (ab178547, Abcam, Cambridge, United Kingdom) and Siah1 (ab2237, Abcam, Cambridge, United Kingdom) were purchased. To evaluate the immunohistochemistry results, the percentage of positive cells was scored from grade 0 to grade 3: Grade 0 (negative), <1% or 0% of the cells were stained; Grade 1 (weak), 1–49% of the cells were stained; Grade 2 (moderate), 50–70% of the cells were stained; and Grade 3 (intense), >70% of the cells were stained (Hotz et al., 2007; Shen et al., 2013; Wang et al., 2016). For immunofluorescence staining, the antibodies used included WISP1 (ab178547, Abcam, Cambridge, United Kingdom), Tp53 (ab16465, Abcam, Cambridge, United Kingdom) and Siah1 (ab2237, Abcam, Cambridge, United Kingdom). Cells were then fixed with 4% Paraformaldehyde and permeabilized with 0.1% Triton X-100. The antibodies were applied at 4°C overnight. FITC-conjugated goat anti-mouse IgG, Rhodamine-conjugated goat anti-rabbit IgG and FITC-conjugated rabbit anti-goat IgG were used as secondary antibodies. Fluorescently-labeled secondary antibodies were added and incubated for 1 h at room temperature. Eventually, nuclei were counterstained with DAPI. The stained cells were mounted on glass slides and examined by confocal microscopy (Wang et al., 2016; Li et al., 2017).

### Statistical Analysis

Statistical analyses were carried out using SPSS 19.0 software (SPSS Inc., Chicago, IL, United States). Each experiment was repeated at least three times. The results were presented as the mean ± standard deviation (SD). Student’s *t*-test and Kruskal–Wallis test were used to assess the statistical significance of differences among the different groups. Postoperative survival was evaluated using the Kaplan–Meier method and log-rank test. Pearson’s correlation test was used for correlation analysis. *P*-value less than 0.05 was statistically considered significant.

## Results

### The Expression and Clinical Significance of WISP1 in PDAC Tissues With *Tp53* Mutation

To determine whether the differential expression of WISP1 was correlated with *Tp53* mutation, immunohistochemistry staining was performed to assess the WISP1 levels in 203 PDAC tissues (including 82 tissues with wild-type *Tp53* and 121 ones with *Tp53* mutation) and 203 paraneoplastic tissues. Samples were scored from grade 0 to 3 as previously described in MATERIALS AND METHODS. For 203 paraneoplastic tissue samples, negative, weak, moderate, or intense WISP1 staining was observed in 87, 80, 31, and 5 tissues, respectively. For 82 PDAC tissues with wild-type *Tp53*, negative, weak, moderate, or intense WISP1 staining was observed in 27, 31, 19, and 5 tissues, respectively.

**FIGURE 1:**
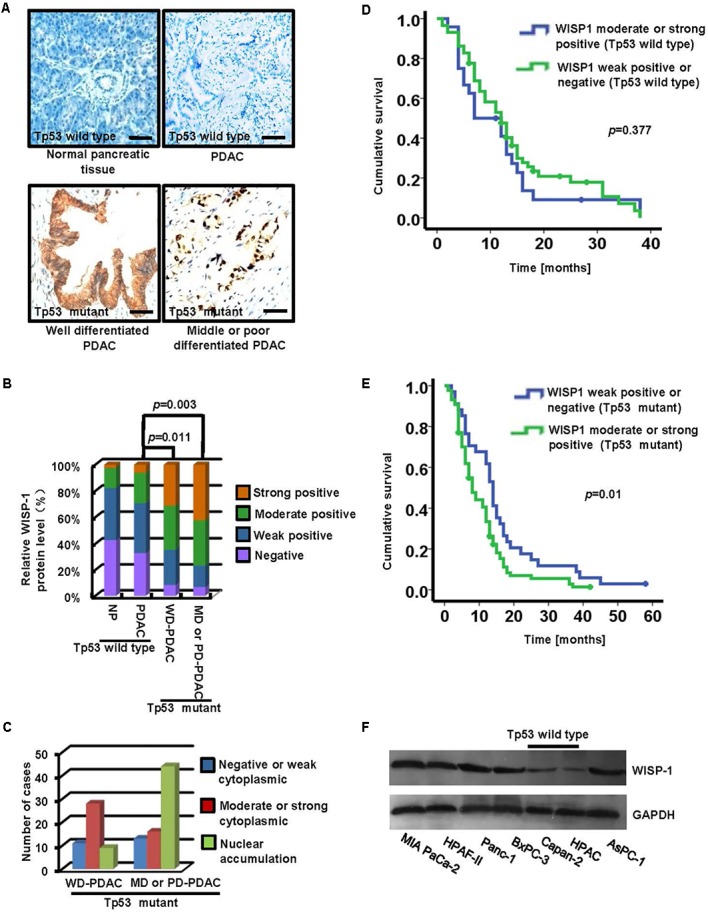
The expression and clinical significance of WISP1 in PDAC with *Tp53* mutation. **(A–C)** Immunohistochemistry staining of WISP1 was summarized in the histogram. WD-PDAC, MD-PDAC, and PD-PDAC represent well, moderately, and poorly-differentiated adenocarcinoma, respectively (black bar scale = 100 μm). **(D)** Kaplan–Meier survival curves for moderate or strong WISP1 staining (Grade 2 or 3) and negative or weak WISP1 staining (Grade 0 or 1) in patients with wild-type *Tp53* were performed using the log-rank test. **(E)** Kaplan-Meier survival curves for moderate or strong WISP1 staining (Grade 2 or 3) and negative or weak WISP1 staining (Grade 0 or 1) in patients with *Tp53* mutation were performed using the log-rank test. **(F)** WISP1 was detected in PDAC cell lines with or without *Tp53* mutation.

In contrast, negative, weak, moderate, or intense WISP1 staining was observed in 4, 13, 16, and 15 of the 48 well-differentiated adenocarcinoma tissues (WD-PDAC) with *Tp53* mutation and in 5, 12, 25, and 31 of the 73 moderately- or poorly-differentiated adenocarcinoma tissues (MD- or PD-PDAC) with *Tp53* mutation (**Figures 1A,B**). In addition, negative or weak WISP1 cytoplasmic staining, moderate or strong WISP1 cytoplasmic staining, and WISP1 nuclear staining were observed in 11, 29, and 8 of the 48 WD-PDAC tissues, and 13, 16, and 44 of the 73 MD or PD-PDAC tissues (**Figure 1C**). These data showed that WISP1 protein levels were significant upregulated in PDAC tissues with *Tp53* mutation than in PDAC tissues with wild-type *Tp53*. In addition, a significant correlation was observed between increased malignant phenotype of tumors from WD-PDAC to MD- or PD-PDAC and shift from cytoplasmic expression to nuclear accumulation of WISP1.

Moreover, the PDAC samples with wild-type *Tp53* or *Tp53* mutation were divided into two groups for clinicopathological evaluation according to the WISP1 levels respectively: moderate or strong WISP1 staining (Grade 2 or 3), and negative or weak WISP1 staining (Grade 0 or 1). Kaplan-Meier analysis showed that the postoperative median survival was 11.78 months for patients with moderate or strong WISP1 staining and 14.51 months for patients with negative or weak WISP1 staining, as illustrated in **Figure 1D** (wild-type *Tp53*) (*P* = 0.377). In contrast, the postoperative median survival was 10.70 months for patients with moderate or strong WISP1 staining and 16.12 months for patients with negative or weak WISP1 staining, as showed in **Figure 1E** (*Tp53* mutation) (*P* = 0.01). These data demonstrated that *WISP1* upregulation may contribute to the poor prognosis in PDAC patients with *Tp53* mutation.

Furthermore, we investigated whether *WISP1* expression was associated with *Tp53* mutation in human PDAC cell lines. The WISP1 protein levels in MIA PaCa-2, HPAF-II and Panc-1, BxPC-3 and AsPC-1 cells with *Tp53* mutation were significantly higher than those in Capan-2 and HPDC cells with wild-type *Tp53* (**Figure 1F**) (*n* = 3).

### *WISP1* Expression Promoted the Malignancy of Cells

**FIGURE 2:**
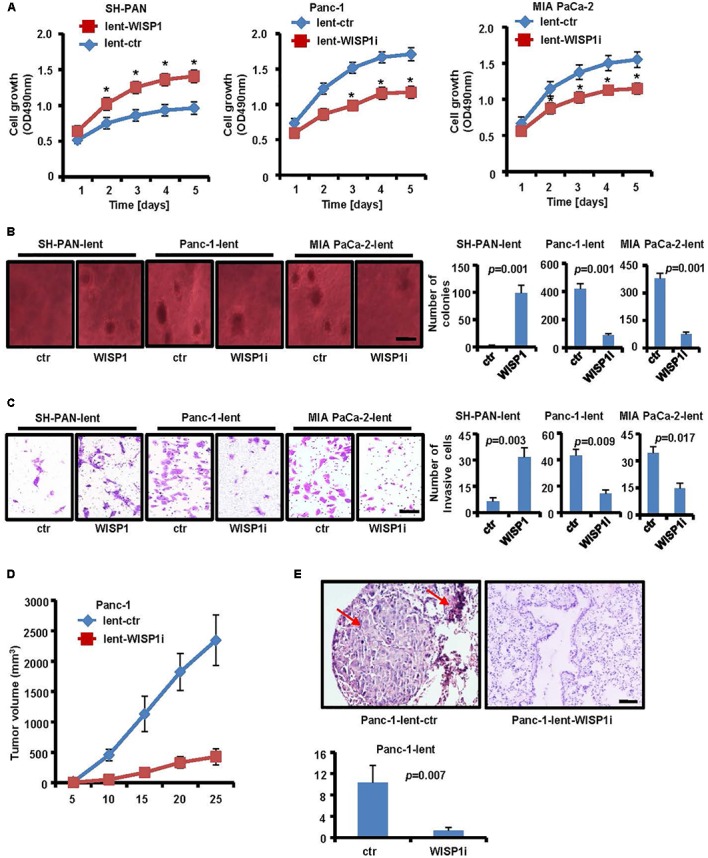
Biological role of *WISP1* in pancreatic carcinogenesis. After lentivirus-mediated WISP1-overexpression vector or control (lent-WISP1 or lent-ctr) was transduced into SH-PAN cells and lentivirus-mediated WISP1-silencing vector or control (lent-WISP1 or lent-ctr) was transduced into PDAC cells, MTT assay **(A)**, soft agar assay **(B)** (black bar scale = 500 μm) and Matrigel invasion assay **(C)** (black bar scale = 50 μm) showed the anchorage-dependent and anchorage-independent growth ability, and tumor invasion rate. The subcutaneous tumorigenic ability **(D)** and lung metastasis after vein injection **(E)** of tumor cells were measured (*n* = 5). HE-stained sections of lung: red arrow, metastatic nodule. ^∗^*P* < 0.05; ^∗∗^*P* < 0.01; ^∗∗∗^*P* < 0.001 (black bar scale = 200 μm).

As the function of *WISP1* in PDAC is unclear, we attempted to determine its biological role. Firstly, lentivirus-mediated mouse *WISP1*-overexpression vector or control was stably transduced into SH-PAN cells (SH-PAN-lent-WISP1 and SH-PAN-lent-ctr) and lentivirus-mediated human *WISP1*-silencing vector or control was stably transduced into Panc-1 cells (Panc-1-lent-WISP1i and Panc-1-lent-ctr) and MIA PaCa-2 cells (MIA PaCa-2-lent-WISP1i and MIA PaCa-2-lent-ctr). The efficiency of *WISP1* overexpression in SH-PAN cell line and *WISP1* knockdown in Panc-1 or MIA PaCa-2 cell line was confirmed by real-time PCR and Western blot analysis (**Supplementary Figure S1**). MTT assay showed that *WISP1* overexpression promoted the growth rate of SH-PAN, and *WISP1* silencing attenuated growth rate of Panc-1 cells and MIA PaCa-2 cells (**Figure 2A**) (*n* = 3). Soft agar assay showed that the SH-PAN-lent-ctr cells did not form colonies while the SH-PAN-lent-WISP1 formed 98.7 ± 14.7 colonies (*P* = 0.001), and the Panc-1-lent-ctr and MIA PaCa-2-lent-ctr cells formed 416.3 ± 37.2 and 378 ± 28.6 colonies respectively (*P* = 0.001), while the Panc-1-lent-WISP1i and MIA PaCa-2-lent-WISP1i cells only formed 89 ± 9.4 (*P* = 0.001) and 76 ± 8.8 (*P* = 0.001) colonies respectively, after 26 days (**Figure 2B**) (*n* = 3). The invasion assay showed that the overexpression of *WISP1* strengthened the invasive ability of SH-PAN (6.3 ± 2 vs. 31.7 ± 5.2 of stained cells, *P* = 0.003), and *WIPS1* silencing attenuated the invasive ability of Panc-1 (43 ± 4.9 vs. 14.3 ± 2.9 of stained cells, *P* = 0.009) and MIA PaCa-2 cells (34.3 ± 3.4 vs. 14.7 ± 3.1 of stained cells, *P* = 0.017) (**Figure 2C**) (*n* = 3). To determine whether *WISP1* plays an important role in the *in vivo* tumorigenicity and micrometastasis of PDAC cells, Panc-1-lent-WISP1i, MIA PaCa-2-lent-WISP1i cells or the control were injected into the subcutaneous tissue or the tail vein of five nude mice each. The results demonstrated that silencing of *WISP1* resulted in a significant decrease in tumorigenicity (*P* = 0.001) (**Figure 2D**). Moreover, quantification of micrometastasis in lung tissues and H&E staining showed that *WISP1* silencing inhibited the *in vivo* metastasis of PDAC cells (10.3 ± 3.21 vs. 1.3 ± 0.58, *P* = 0.007) (**Figure 2E**). These data suggest that *WISP1* acted as a potential oncogene in PDAC cells.

### *Tp53* Mutation May Promote *WISP1* Expression in SH-PAN and PDAC Cells

**FIGURE 3:**
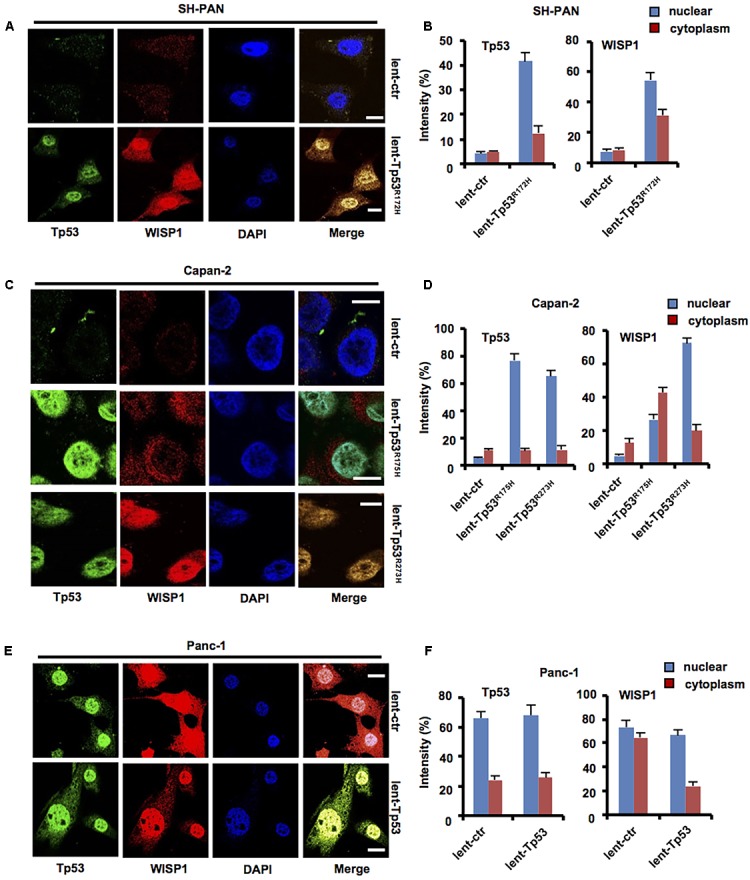
*Tp53* mutation may promote *WISP1* expression in SH-PAN and PDAC cells. After lentivirus-mediated mouse *Tp53^*R172H*^* overexpression vector or control was stably transduced into SH-PAN cells, lentivirus-mediated human *Tp53^*R175H*^* or *Tp53^*R273H*^* overexpression vector or control was stably transduced into Capan-2 cells (*Tp53* wild-type), lentivirus-mediated human wild-type *Tp53* overexpression vector or control was stably transduced into Panc-1 cells (*Tp53* mutation), and then immunofluorescence staining was performed to measure the levels and locations of WISP1 in SH-PAN **(A,B)**, Capan-2 **(C,D)**, and Panc-1 cells **(E,F)** (white bar scale = 10 μm).

To determine whether *WISP1* expression was affected by *Tp53*, lentivirus-mediated mouse *Tp53^*R172H*^* overexpression vector or control was stably transduced into SH-PAN cells, lentivirus-mediated human *Tp53^*R175H*^* or *Tp53^*R273H*^* overexpression vector or control was stably transduced into Capan-2 cells (wild-type *Tp53*), lentivirus-mediated human wild-type *Tp53* overexpression vector or control was stably transduced into Panc-1 cells (*Tp53* mutation), and then immunofluorescence staining was performed to measure the levels and locations of WISP1 in SH-PAN, Capan-2, and Panc-1 cells. The results showed that nuclear and cytoplasm WISP1 staining was sharply enhanced with overexpression of *Tp53^*R172H*^* in SH-PAN cells (**Figures 3A,B**) and with overexpression of *Tp53^*R175H*^* or *Tp53^*R273H*^* in Capan-2 cells (**Figures 3C,D**) (*n* = 3). In addition, the cytoplasm WISP1 staining was sharply enhanced with overexpression of wild-type Tp53 in Panc-1 cells, however, the nuclear WISP1 staining was not affected (**Figures 3E,F**) (*n* = 3). These data suggest that *Tp53* mutation may promote *WISP1* expression in SH-PAN cells and PDAC cells.

### *Siah1* May Inhibit *WISP1* Expression in PDAC Cells

As previous study showed that wild-type *Tp53* may promote Siah1 expression which may mediate ubiquitination degradation of downstream target gene, we attempted to investigate whether *WISP1* expression was correlated with *Siah1*.

**FIGURE 4:**
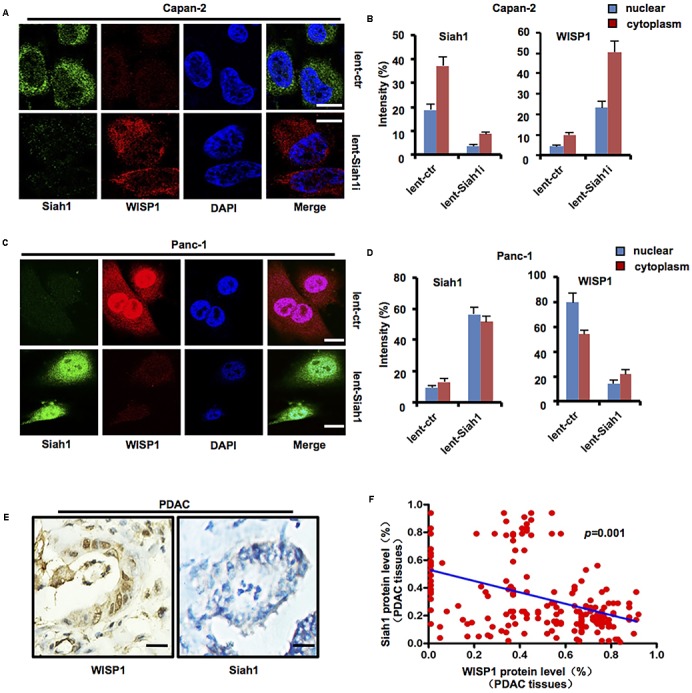
*Siah1* may inhibit *WISP1* expression in PDAC cells. After lentivirus-mediated Siah1 silencing vector or control was stably transduced into Capan-2 cells (*Tp53* wild-type), lentivirus-mediated Siah1 overexpression vector or control was stably transduced into Panc-1 cells (*Tp53* mutation), and then immunofluorescence staining was performed to measure the levels and locations of Siah1 and WISP1 in Capan-2 **(A,B)** and Panc-1 cells **(C,D)** (white bar scale = 10 μm). Immunohistochemistry staining of Siah1 and WISP1 was performed, and Pearson’s correlation test was also used to analyze the correlation between Siah1 and WISP1 according to immunohistochemistry staining **(E,F)** (black bar scale = 25 μm).

Lentivirus-mediated Siah1 silencing vector or control was stably transduced into Capan-2 cells (wild-type *Tp53*), lentivirus-mediated Siah1 overexpression vector or control was stably transduced into Panc-1 cells (*Tp53* mutation), and immunofluorescence staining was performed to measure the levels and locations of Siah1 and WISP1 in Capan-2 and Panc-1 cells. The results showed that Siah1 staining was mainly focused on the cytoplasm in PDAC cells, and nuclear and cytoplasm WISP1 staining was sharply enhanced with silencing of *Siah1* in Capan-2 cells (**Figures 4A,B**) (*n* = 3), however, nuclear and cytoplasm WISP1 staining was sharply downregulated with overexpression of *Siah1* in Panc-1 cells (**Figures 4C,D**) (*n* = 3). Moreover, to testify whether Siah1 showed a negative correlation with WISP1 in 203 PDAC tissue samples, the Pearson’s correlation analysis was conducted. The results showed that Siah1 expression was significantly and negatively correlated with WISP1 expression in 203 PDAC tissue samples (**Figures 4E,F**).

The above-mentioned data suggest that *Siah1* may inhibit *WISP1* expression in PDAC cells.

### *Tp53* Mutation May Inhibit Siah1-Mediated WISP1 Ubiquitination Degradation

**FIGURE 5:**
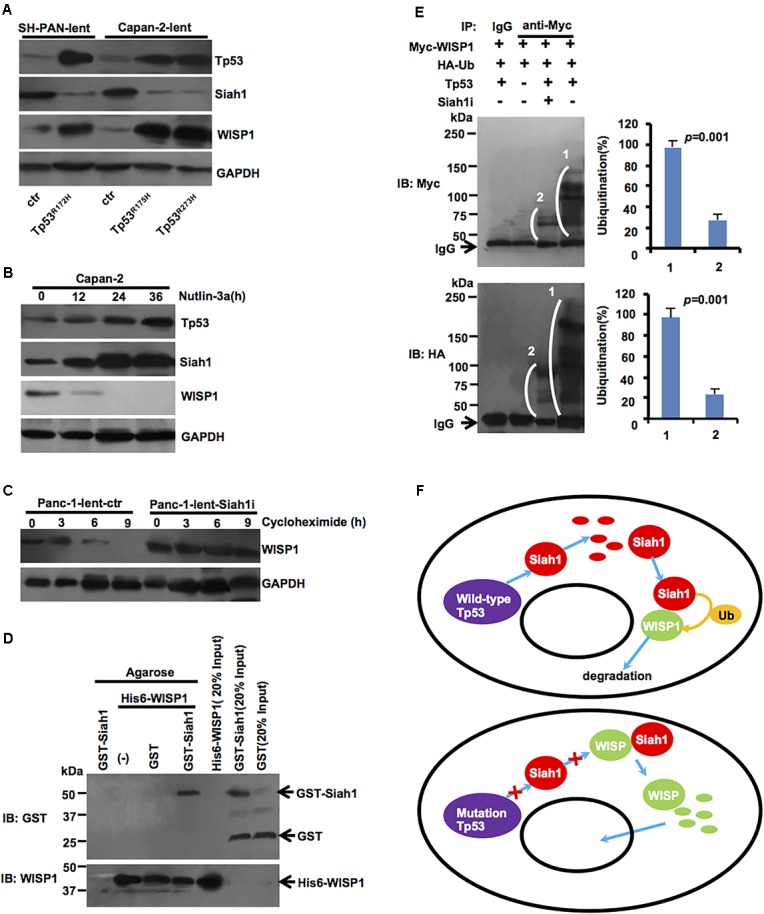
*Tp53* mutation may inhibit ubiquitination degradation of Siah1-mediated WISP1. **(A)** After lentivirus-mediated mouse *Tp53^*R172H*^* overexpression vector or control was stably transduced into SH-PAN cells, lentivirus-mediated human *Tp53^*R175H*^* or *Tp53^*R273H*^* overexpression vector or control was stably transduced into Capan-2 cells, Western blot analysis was performed to validate Siah1 and WISP1 levels. **(B)** Capan-2 cells were treated with 10 μM of Nutlin-3a at defined time period and were examined by immunoblotting. **(C)** Panc-1 cells transduced with lentivirus-mediated Siah1 silencing vector or control were treated with 100 μg/ml of cycloheximide (CHX) at defined time periods and were examined for WISP1 expression by Western blot. **(D)** His6-WISP1 was immobilized on Ni-NTA magnetic agarose beads (columns 2–4) and incubated with GST-Siah-1 (column 4), GST alone (column 3) or without additional protein (column 2). In column 1, GST-Siah-1 was incubated with Ni-NTA magnetic agarose beads without His6-tagged WISP1. After extensive washing, the beads were boiled in SDS sample buffer and the eluted proteins were analyzed by immunoblotting using anti-GST and anti-WISP1 antibodies. In columns 5, 6, and 7, His6-WISP1, GST-Siah-1, and GST alone were run directly as input controls. **(E)** Myc-tagged WISP1, HA-tagged ubiquitin (Ub), and full-length Tp53 were transiently expressed in Capan-2 cells, as indicated, which were pre-treated with control or Siah-1 shRNA. After treatment with MG132, protein lysates were prepared, immunoprecipitated with anti-Myc antibody or control IgG, and then analyzed by Western blot using anti-Myc antibody (upper) and anti-HA antibody (lower). White brackets indicate smear signals showing poly-ubiquitination. The strong signals at the bottom of the upper image were corresponded to IgG heavy chains. **(F)** Simplified schematic diagram of the Tp53-Siah1-WISP1 pathway in PDAC cells (wild-type *Tp53* or *Tp53* mutation). Wild-type *Tp53* may promote Siah1 protein levels, which may act as an E3 ubiquitin-protein ligase that mediates ubiquitination and subsequent proteasomal degradation of WISP1. Meanwhile, *Tp53* mutation may downregulate Siah1 protein levels, which may inhibit ubiquitination and degradation Siah1-dependent WISP1, and induce WISP1 nuclear import.

To further determine whether *Tp53* mutation may affect the expression of Siah1 or WISP1, lentivirus-mediated mouse *Tp53^*R172H*^* overexpression vector or control was stably transduced into SH-PAN cells, and lentivirus-mediated human *Tp53^*R175H*^* or *Tp53^*R273H*^* overexpression vector or control was stably transduced into Capan-2 cells. Western blot analysis showed that *Tp53* mutation downregulated the Siah1 level and upregulated WISP1 level in SH-PAN cells and Capan-2 cells (**Figure 5A**) (*n* = 3). Also, the stabilization and activation of endogenous Tp53 by the small-molecule inhibitor of MDM2 (Nutlin-3a) (10 μM) resulted in an increase in Siah1 and a decrease in WISP1 expression (**Figure 5B**) (*n* = 3). Moreover, the inhibition of protein synthesis by cycloheximide, followed by Western blot at different time points, showed that Siah-1 silencing remarkably maintained the protein level of WISP1 (**Figure 5C**) (*n* = 3). To determine whether Siah1 protein may directly bind to WISP1 protein, a GST-Siah-1 fusion protein and a His-tagged WISP1 protein were synthesized by *in vitro* transcription and translation and used in an immunoprecipitation-immunoblot experiment, Siah-1 was shown to directly interact with WISP1 *in vitro* (**Figure 5D**) (*n* = 3). Then, all the data described above motivated us to examine whether Siah-1 is an E3 ubiquitin-protein ligase that mediates ubiquitination and subsequent proteasomal degradation of WISP1. A transient expression-based assay was performed in which exogenously expressed Tp53, WISP1 (Myc-tagged), and ubiquitin (HA-tagged) induced *de novo* WISP1 ubiquitination detected by anti-Myc antibody or anti-HA antibody in wild-type *Tp53* in Capan-2 cells. The inhibition of Siah-1 was accomplished with lentivirus-mediated Siah1 silencing vector which nearly completely abrogated the Tp53-dependent signals detected by anti-Myc antibody (corresponding to the ubiquitination of Myc-tagged WISP1 alone) and by anti-HA antibody (**Figure 5E**) (*n* = 3). Taken together, these data indicate that Siah-1 is essential to Tp53-induced WISP1 ubiquitination.

A simplified schematic diagram of the genetic pathway is illustrated in **Figure 5F**.

## Discussion

Pancreatic cancer, with an overall 5-year survival rate of < 9%, is the fourth leading cause of cancer death in the past few years, which has the worst prognosis of any major malignancy (Siegel et al., 2018). Compelling data have reported that some major suppressors are involved in pancreatic carcinogenesis, such as *p16*, *Tp53* and *Smad4*. However, the vehicle that permits early diagnosis or effective treatment for pancreatic cancer is limited. *Tp53* is one of the most well-known pancreatic cancer suppressors that is inactivated in approximately 50–75% of pancreatic cancers. Alterations of Tp53 protein function permits cells to bypass DNA damage checkpoints and apoptotic signals (Rosenfeldt et al., 2013). Evidence is accumulating that loss of Tp53 function may impose genomic instability in pancreatic cancers (Su et al., 2002; Mello et al., 2017). *Trp53*^*R172H*^ mutation cooperates with *Kras*^*G12D*^ to promote chromosomal instability and widely metastatic PDAC in mice (Hingorani et al., 2005). Tp53 may have a crosstalk with Wnt signaling. Wild-type Tp53 has been found to inhibit Wnt signaling by different mechanisms including the induction of microRNA-34 (Kim et al., 2011). Loss of Tp53 function increases canonical Wnt signaling through miR-34-specific interactions with target UTRs, whereas miR-34 depletion relieves p53-mediated Wnt repression. Loss of Tp53 or miR-34 contributed to neoplastic progression by triggering the Wnt-dependent, and tissue-invasive activity of colorectal cancer cells. Furthermore, during development, miR-34 interactions with the β-catenin UTR determine Xenopus body axis polarity and Wnt-dependent gene patterning (Sadot et al., 2001; Levina et al., 2004).

To further perceive the downstream molecular alteration in the course of *Tp53* mutation in pancreatic carcinogenesis, microarray has been used to explore differentially expressed by genes which may act as potential therapeutic targets. Our previous study has also demonstrated that *Tp53* mutation may promote WISP1 expression in mouse PDAC cells (Wang et al., 2015). Recent studies had shown that WISP1 acts as a new oncogene in glioblastoma (Jing et al., 2017), oral squamous cell carcinoma (Jung et al., 2017), gastric cancer (Jia et al., 2017), melanoma (Shao et al., 2016), and colon cancer (Wu et al., 2016). Also, Yang et al. (2015) showed that patients with high expression of WISP-1 had a shorter survival time independent of clinical stage and lymphatic metastasis status in pancreatic cancer. However, the biological function and underlying mechanism of WISP1 in pancreatic carcinogenesis still remains unclear. In our study, we interestingly found that WISP1 protein level was more significantly upregulated in PDAC tissues with *Tp53* mutation than in PDAC tissues with wild-type *Tp53*. And a significant correlation was observed between increased malignant phenotype of tumors from WD-PDAC to MD- or PD-PDAC and shift from cytoplasmic expression to nuclear accumulation of WISP1. To identify the role of WISP1 in PDAC, its clinical significance and biological function were investigated. Surprisingly, Kaplan–Meier analysis showed that WISP1 expression was probably correlated with the poor prognosis in PDAC patients with only *Tp53* mutation. Also, biological behavior analysis showed that *WISP1* may promote proliferation, invasion, tumorigenicity, and micrometastasis of mouse PanIN cells and human PDAC cells, which indicated that WISP1 may act as a potential oncogene in pancreatic carcinogenesis. Moreover, *Tp53* mutation may promote *WISP1* expression in mouse PanIN cells as well as human PDAC cells.

In our study, we showed that *Tp53* mutation may contain high expression of *WISP1*, while “*Tp53* mutation” and “*WISP1* high expression” presented a close correlation in pancreatic carcinogenesis. Su et al. (2002) showed that WISP1 acts to block cell death at a late-stage in the p53-mediated apoptosis pathway. However, the regulatory mechanism of how *Tp53* mutation affects *WISP1* expression still remains unclear.

It is well-known that Siah1 encodes a protein that is a member of the seven in absentia homolog (SIAH) family. Besides, the protein is an E3 ligase which is involved in ubiquitination and proteasome-mediated degradation of specific proteins. Ji et al. (2017) showed that Wnt-induced dissociation of the Axin/GSK3 complex permits Siah to interact with Axin which is not associated with GSK3, and promotes Axin degradation mediated by Siah, which represents an important feed-forward mechanism to achieve sustained Wnt/β-catenin signaling. In our study, the data showed that *Tp53* mutation inhibited *Siah1* expression, which inhibited ubiquitination and degradation of WISP1 mediated by Siah1.

## Conclusion

In summary, our findings show that WISP1 expression was correlated with the poor prognosis in PDAC patients with *Tp53* mutation, and WISP1 may act as a potential oncogene in PDAC cells. *Tp53* mutation downregulated Siah1 protein levels and further inhibited ubiquitination and degradation of Siah1-dependent WISP1 and induced WISP1 nuclear import, which may be a new signaling pathway playing an important role in pancreatic carcinogenesis.

## Author Contributions

WW, XL, LmW, and TL contributed in data acquisition, data analysis, and interpretation, as well as drafting of manuscript. YZa, YQ, and TB conducted data acquisition, technical support, and statistical analysis. JL and MX conducted cancer case-control study. YZh, QW, and LfW contributed in study design, data analysis and interpretation, drafting of manuscript, study’s supervision, and critical revision of the manuscript for important intellectual content.

## Conflict of Interest Statement

The authors declare that the research was conducted in the absence of any commercial or financial relationships that could be construed as a potential conflict of interest.

## Supplementary Material

FIGURE S1 Real-time PCR and Western blot analysis confirmed the efficiency of *WISP1* overexpression in SH-PAN cell line and *WISP1* knockdown in Panc-1 or MIA PaCa-2 cell line.

## Abbreviations

MD-PDAC: moderately-differentiated pancreatic ductal adenocarcinoma
PanIN: pancreatic intraepithelial neoplasia
PDAC: Pancreatic ductal adenocarcinoma
PD-PDAC: poorly-differentiated pancreatic ductal adenocarcinoma
WD-PDAC: well-differentiated pancreatic ductal adenocarcinoma
WISP1: Wnt1 inducible signaling pathway protein-1
