# Supplementary figures and images for: Tp53 Mutation Inhibits Ubiquitination and Degradation of WISP1 via Down-Regulation of Siah1 in Pancreatic Carcinogenesis
Source: Front Pharmacol. 2018 Aug 3;9:857. doi: 10.3389/fphar.2018.00857 (PMC6085464; doi:10.3389/fphar.2018.00857)

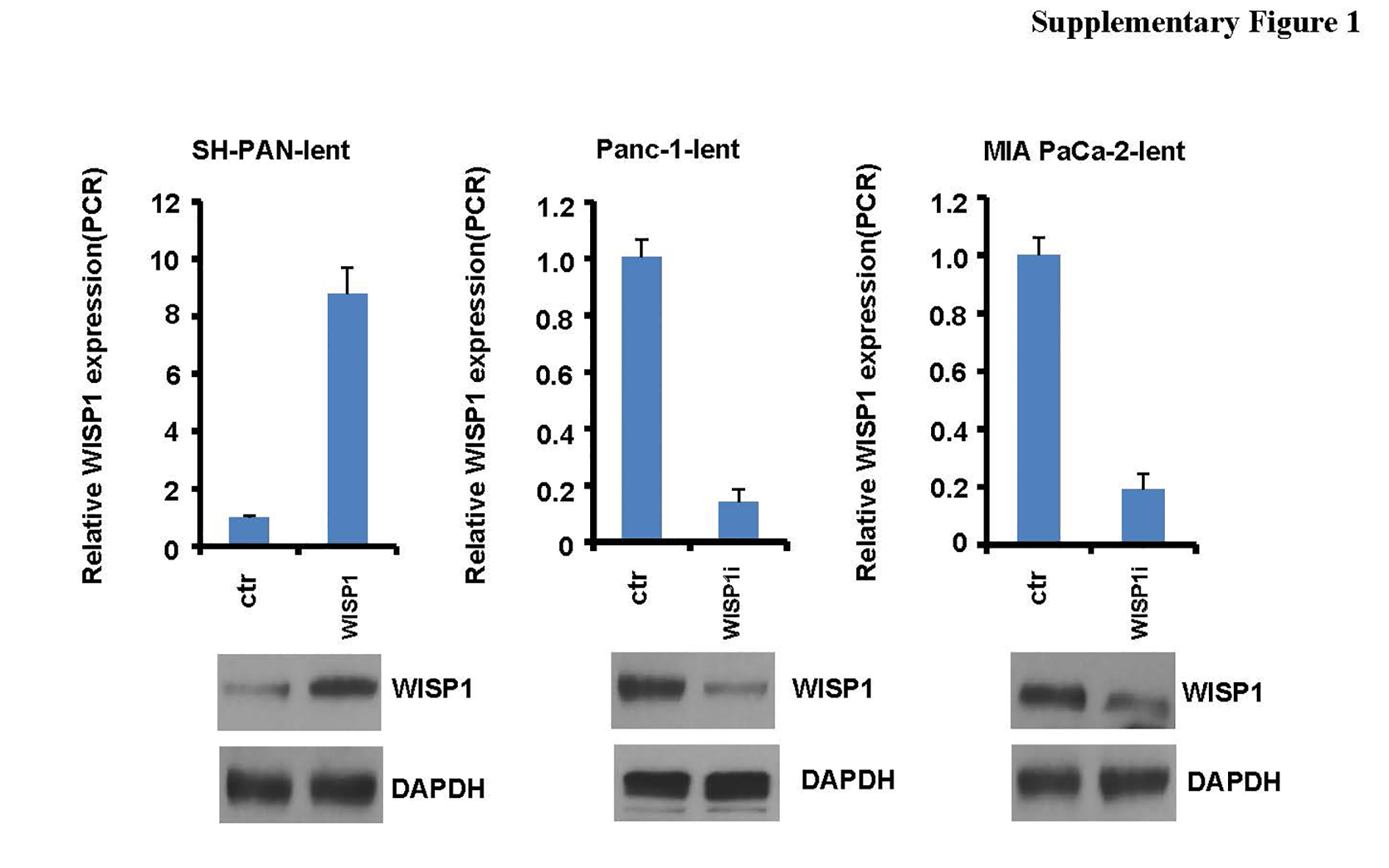

Supplement: FIGURE S1 — Real-time PCR and Western blot analysis confirmed the efficiency of WISP1 overexpression in SH-PAN cell line and WISP1 knockdown in Panc-1 or MIA PaCa-2 cell line. [file Image_1.JPEG]
